# Supplementary material for: Are carbon emissions trading and green financial instruments synergistic? -Comprehensive quantitative research based on content analysis
Source: PLoS One. 2024 Mar 7;19(3):e0298601. doi: 10.1371/journal.pone.0298601 (PMC10919601; doi:10.1371/journal.pone.0298601)
Supplement: S1 Data — (ZIP) [file pone.0298601.s001.zip › Data/地级市Z_F工作报告.zip!/║╙─╧/┐¬╖Γ╩╨/└┤╫╘╣1⁄2╓┌║┼í╛┬φ┐╦╩2╛▌═°í┐_2010.docx]

各位代表：

现在，我代表市人民政府，向大会作政府工作报告，请予审议，并请各位政协委员和列席人员提出意见。

一、2009年工作回顾2009年是新世纪以来我市经济社会发展最为困难、受到冲击最为严重的一年，也是我们经受严峻考验、在逆境中奋力跨越的一年。一年来，在省委、省政府和市委的坚强领导下，全市上下深入贯彻落实科学发展观，积极应对国际金融危机，齐心协力破危局，集中精力谋发展，全面完成了市十三届人大一次会议确定的各项目标任务。

——经济实现平稳较快发展。全市生产总值777.1亿元，增长12.6%；财政一般预算收入29.5亿元，增长13.1%，“双11”增长目标超额完成。农业生产战胜了新中国成立以来最严重的旱灾，粮食总产50.7亿斤，连续六年保持增长；文化旅游产业继续保持快速发展势头。经济企稳回升态势好于预期。

——跨越式发展良好态势得以保持。第三产业增加值、城镇固定资产投资、规模以上工业增加值、市本级财政一般预算收入、社会消费品零售总额、财政一般预算收入等主要经济指标增幅分别位居全省第一、第二、第三、第四、第五和第六位。经济发展速度连续两年超过全省平均水平。

——招商引资实现重大突破。全市共引进3000万元以上项目333个，到位资金175亿元；其中引进省外资金141亿元，增长22%，增幅居全省第四位。特别是引进了奇瑞汽车、河南煤化重装集团、开封新区基础设施BT项目等一批重大项目，必将对开封经济社会发展起到巨大的推动作用。

——重点项目建设取得新进展。全市209个重点项目完成投资205.6亿元，是2008年的1.6倍；其中120个工业项目完成投资126亿元。京源公司2×600MW发电机组、晋开集团合成氨净化系统改造等一批重大项目建成投产。全市城镇固定资产投资315.6亿元，增幅达到35.8%，危机之年继续保持了高位增长。

——民生得到进一步改善。在财政十分紧张的情况下，落实资金25.6亿元，圆满完成15件为民实事。全市城镇居民人均可支配收入12318元，增长8.6%；农民人均纯收入4695元，增长7.8%。社会保障水平明显提高；教育、卫生、文化、体育等各项社会事业全面发展。

——社会大局保持和谐稳定。认真办理人民群众来信来访，及时化解人民内部矛盾，妥善处置各类突发事件，严厉打击各种刑事犯罪，人民群众安全感明显提高；认真落实安全生产责任制，安全生产形势总体平稳，全市社会大局和谐稳定。

一年来，我们主要做了以下工作：

（一）全力应对国际金融危机，保持经济平稳较快发展。面对国际金融危机对我市经济发展带来的严重影响，坚持把保持经济平稳较快发展、保持经济社会跨越式发展良好态势作为全市经济工作的首要任务，确立了“扩内需、促增长、调结构、抓项目、保态势”的总体思路，着力增强工作的前瞻性、预见性，及时把握不同时段经济运行的突出矛盾和问题，出台实施了促进经济增长“60条措施”，多次召开经济运行会议安排部署，强化工作指导；抓住市场开拓、企业服务、银企对接、投资拉动、招商引资、产业集聚区建设等关键环节，深入开展“企业服务年”、“百个亿元项目大招商”等活动。一系列应对举措最大限度地减轻了危机对我市的不利影响，迅速遏制了经济下滑，加快了经济企稳回暖势头，全市经济呈现出“一季度触底、二季度回升、下半年向好”的发展态势，实现了平稳较快发展的目标。

（二）强力推进工业强市战略，工业经济实力不断增强。坚持把保工业作为战危机、保增长的重点，着力加强企业服务，狠抓重点企业培育，积极推进企业上市，工业经济继续保持平稳较快发展。全市工业增加值319.3亿元，增长13%，其中规模以上工业增加值220.7亿元，增长17.8%。扎实开展企业服务年活动。确定了113户市重点服务企业和686户县区重点服务企业，出台了一系列政策措施，帮助企业解决实际困难；多次召开银企洽谈会，落实贷款173亿元，全市金融机构新增贷款84.2亿元，较年初增长34.4%，是上年增量的3.7倍；着力抓好企业产销对接，签约金额31.7亿元；加快中小企业信用担保体系建设，为中小企业直接融资近10亿元。狠抓重点企业培育。实施“双30计划”，对30户重点工业企业和30个产业升级项目在政策和生产要素等方面给予重点倾斜，30户重点工业企业实现主营业务收入161.9亿元，增长18.3%。全市规模以上工业企业达到1178家，比2008年年底增加210家；其中主营业务收入超亿元企业189家。积极推进企业上市。“中阀科技”在美国纳斯达克主板市场成功上市；德意环保在美国成功路演。

（三）切实抓好“三农”工作，农业农村经济全面发展。紧紧围绕“稳粮、增收、强基础、重民生”的目标，毫不松懈地抓好农业生产，千方百计增加农民收入，保持了农业农村发展好形势。认真落实各项惠农政策。发放各项惠农补贴7亿元；提高粮食最低收购价标准，每斤平均提高0.11元；扩大家电下乡补贴品种，启动了汽车（摩托车）下乡，补贴资金8827万元。着力增强农业综合生产能力。狠抓农田水利基本建设，改善灌溉面积84.3万亩；提高农业机械化装备水平，农业机械化率达到67%；全力组织抗旱斗争，夺取了抗旱夺丰收决定性胜利，粮食生产再获丰收。大力发展农业产业化经营。积极引进培育龙头企业，全市省级农业产业化龙头企业增加到10个；鼓励发展各类农民合作经济组织，农民专业合作社发展到1002家；积极推进畜牧产业化，全市标准化养殖小区达到486个，规模养殖场（户）超过4万个；肉、蛋、奶总产分别增长5.5%、11.5%和12.6%。不断改善农村生产生活条件。扎实推进社会主义新农村建设，完成了1200个行政村村庄规划；改建农村公路786公里，解决了28.7万农村群众饮水安全问题，发展农村户用沼气3.5万户，新建农村连锁超市432个，改造农村卫生厕所3500座；完成扶贫开发整村推进村59个，脱贫人口6万人；累计完成106个示范村和637个整治村村容村貌整治，农村面貌明显改善。

（四）大力发展文化旅游业，第三产业支撑能力显著提升。以创建国家级文化产业示范园区为契机，以促进文化旅游产业融合发展为重点，以实施文化旅游产业项目建设为抓手，不断加快文化旅游业发展步伐，有力带动了第三产业发展。着力办好重大文化旅游节会活动。精心谋划，认真组织，成功举办了“我们的节日—中国开封2009清明文化节”、中国开封第27届菊花花会暨第二届中国收藏文化（开封）论坛、“金秋汴梁游”、“中原文化宝岛行”等一系列重大文化旅游活动，推动了节会经济的发展。加大文化旅游项目建设力度。抓住国家实施适度宽松货币政策的机遇，深入实施宋都古城风貌保护与重现工程，贯通了水系二期工程，启动了城墙保护四期工程、繁塔·禹王台景区综合开发、大宋文化博物馆·开封城摞城新郑门遗址、刘青霞故居综合开发、延庆观玉皇阁顶升等一批重点文化旅游项目。深入实施旅游带动战略。出台了一系列促进旅游消费的政策措施，不断加大旅游推介力度，旅游业继续保持快速发展势头，全市旅游接待量和旅游综合收入分别增长22.4%和29.6%。文化旅游业的快速发展，有效带动了第三产业发展，全市第三产业增加值增长17.1%，增幅高于全省6.2个百分点，拉动全市经济增长5.7个百分点。

（五）加强基础设施建设，开封新区发展步伐明显加快。紧紧抓住省委、省政府全力打造“郑汴新区”，建设全省经济社会发展核心增长极的重大机遇，完善组织机构，创新体制机制，强力推进开封新区开发和建设。进一步完善规划编制。通过国际招标，编制了郑汴新区空间发展战略规划和总体规划；完成了开封新区中心区规划和修建性详规、职教园区建设规划以及新区基础设施、水系、管网、综合交通等专项规划。启动了《开封新区产业发展总体规划》编制，汽车及零部件产业园、空分特色产业园等产业建设规划基本完成。加大基础设施投入力度。成立了新区基础设施建设投资公司，融资近16亿元。投入12.7亿元，实施了新区一期、二期18条道路建设。总投资过百亿元的郑汴物流通道、黑岗口调蓄水库、连霍高速开封段拓宽、郑民高速开封段、郑州至开封城际铁路等重大基础设施项目相继开工建设。积极推进项目落地。河南煤化集团大型空分设备、铁塔橡胶工业园、郑开·森林半岛等项目建设进展顺利，奇瑞汽车项目正式启动，大河动漫城、河南移动公司生产楼、河南煤化集团研发中心等一批项目前期准备工作基本就绪。首批入驻职教园区的5所学校陆续开工建设。扎实推进迁村并居。闫砦、毛砦迁村并居工程进展顺利，瞿家砦、野厂迁村并居工程正式启动。开封新区被评为“中国最具投资潜力省级十强开发区”。

（六）不断深化改革开放，经济发展活力持续增强。抓住影响我市经济社会发展的主要矛盾，深入推进重点领域和关键环节改革，努力提高对外开放水平，不断为经济社会发展增活力、添动力。强力推进国有企业改革。开封缝纫机总厂、麦芽厂、毛条厂、针织厂破产清算基本结束；开化集团、联合收割机厂、柴油机厂依法破产，职工安置工作接近尾声；开封农药厂关闭重组进入职工安置阶段；市棉麻总公司完成整体改制。扎实推进事业单位改革。积极推进文化旅游体制改革，完成了32家公益性文化事业单位内部三项改革和文艺院团、电影公司、电影院改革；组建了文化市场综合执法机构；筹建了市文化旅游投资集团有限公司。教育、卫生等事业单位实行了全员聘用和绩效工资制度。不断深化行政管理体制改革。新一轮政府机构改革全面启动；投资项目代理制进一步完善；涉企收费“一费制”正式实施；行政审批进一步规范，经济发展环境不断优化。大力开展招商引资。组织参加了豫港投洽会、第四届中博会、第五届河南国际投洽会、第四届豫商大会、中原文化澳洲行、港澳行等重大经贸活动，扎实开展“承接产业转移引进百个亿元大项目”活动，签约亿元以上项目112个，总投资299.5亿元。签约项目数量和总投资额均为历年最多。

（七）着力转变发展方式，促进经济社会可持续发展。把加快构建自主创新体系和环境保护作为转变发展方式的重要抓手，着力推进节约集约发展和可持续发展。加快构建自主创新体系。市级以上工程技术研究中心达到15家，省级工程技术研究中心增加到5家；茂盛机械有限公司被确定为“国家粮食加工装备工程技术研究中心”，实现了我市国家级工程技术中心零的突破。加强与中国农业大学、河南大学等院校的合作，开展技术合作项目30个，市校共建取得新成效。扎实推进节能减排工作。淘汰落后产能企业5家，淘汰5000吨标煤以上企业落后设备368台（套），全市单位GDP能耗下降5%；化学需氧量提前一年完成省下“十一五”污染减排目标。积极推进公共机构节能工作，实施绿色照明工程，推广节能灯34.5万只。加强土地资源管理调控，完成土地整理17.4万亩，连续11年保持耕地总量动态平衡。加强环境保护和生态建设。启动了生活垃圾焚烧发电项目，关停、取缔“十五小”企业16家，关闭不符合国家产业政策企业5家，规范排污口32个。全市所有出境河流断面水质考核指标均达到省下目标要求，饮用水源地水质平均达标率继续保持100%。全年空气环境质量优良天数达到335天，优良率91.8%，大气环境质量明显改善。实施生态惠农工程，创建省级生态文明村12个、省级环境优美小城镇3个。扎实推进林业生态市建设，造林18.3万亩，绿化村庄949个。

（八）着力保障和改善民生，和谐社会建设取得新成效。坚持把改善民生作为工作的出发点和落脚点，围绕办好15件为民实事，加大公共支出，改善公共服务，解决了一批人民群众最关心、最直接、最现实的利益问题。加强就业和再就业工作。全年城镇新增就业6.5万人，实现下岗失业人员再就业2.6万人，其中就业困难人员就业0.9万人；动态消除“零就业家庭”。培训农村劳动力15.1万人，其中免费培训4万人；全年输出劳动力81.5万人。健全社会保障体系。提高城市低保、农村低保和农村五保集中供养标准，基本实现应保尽保；全面推行新型农村合作医疗制度，新农合参保率达到93.28%；投入2830.7万元，解决3.8万名困难企业退休职工医疗保险问题；投入944万元，为1.1万名白内障患者免费实施复明手术；加大城乡医疗救助力度，救助困难群众25.4万人次，发放医疗救助资金1558万元；认真落实“四免一关怀”政策，发放艾滋病致孤及单亲人员救助资金147万元；有效开展救灾救济，救济人员17.9万人次。加大保障性住房建设力度。开工建设经济适用住房61.4万平方米，竣工51.5万平方米，全市具备购买条件的家庭一半以上购买到经济适用住房。开工新建和收购廉租住房2274套；为1.5万户低收入住房困难家庭发放租房补贴3342.7万元。大力发展文化教育体育事业。实施文化惠民工程，新改建11个乡镇综合文化站、230个农家书屋和209处农民体育健身工程；提高义务教育保障水平，农村中、小学义务教育学校生均公用经费标准分别提高到500元和300元；推进职业教育攻坚计划，开封大学顺利通过省示范性高等职业院校中期检查，新校区建设全面启动；成功举办了第三届郑开国际马拉松赛、“全民健身月”等群众性体育活动，全民健身运动深入开展。不断改善医疗卫生条件。市中医院病房楼主体工程竣工；完成了26个薄弱乡镇卫生院改造，改建社区卫生服务中心5个、卫生服务站16个、标准化村卫生室514个、乡镇计生服务站28个；积极应对重大突发传染性疾病的挑战，手足口病、甲型H1N1流感等疾病防控取得良好效果。认真落实计划生育政策。全面实施计划生育家庭特别扶助制度，计划生育奖励扶助标准提高到每人每年840元；扎实开展出生性别比和违法生育专项治理，人口自然增长率控制在4.63‰。切实加强城市建设与管理。黄河大街南延、夷山大街南延工程建成通车，集英街北延、郑汴路改造等建设工程进展顺利。组建了宋都古城建设投资有限公司，融资8亿元启动了小李庄安置用房及老城区1号、2号地块改造前期工作。投入6700多万元，建成了智能交通指挥控制中心和城市报警与监控系统；投入1000万元，新增加58台公交车，调整6条公交线路；投入1048万元，新建水冲式公厕10座，改造老城区旱厕135座。深入开展“创三城”活动，城市居住环境进一步改善。着力构建和谐社会。深入推进社会治安综合治理工作向纵深发展，积极开展机关干部下访、信访积案化解、“警民一家亲”等活动，平安开封建设取得新成效。严厉打击各种违法违规生产活动，深入开展食品药品安全专项整治。切实加强社区建设，推进基层民主政治建设，顺利完成127个社区居委会换届选举工作。加强精神文明建设，公众道德水平和城乡文明程度明显提升。拥军优属、拥政爱民工作取得新进展，军政军民团结进一步巩固，荣获全国全民国防教育先进单位。全面贯彻党的民族宗教政策，各民族和睦相处、和衷共济、和谐发展。

过去的一年，我们坚持不断强化政府自身建设。认真开展深入学习实践科学发展观、“讲党性修养、树良好作风、促科学发展”和大力弘扬焦裕禄精神等活动，持续推进“两转两提”，各级政府机关服务效能和工作水平进一步提升。坚持依法行政，自觉接受人大及其常委会监督，支持政协参政议政，认真办理人大代表议案、建议和政协提案。积极推进政府廉政建设，强化财政监察审计监督，政府系统反腐倡廉工作取得新成效。同时，外事、侨务、对台工作进一步加强，统计、档案、史志、气象、地震、人防、社会科学等工作都取得了新的成绩。对口援建江油工作进展顺利。

各位代表，2009年是新世纪以来经济形势最为复杂、发展最为艰难的一年，开封经济社会发展取得这样的成绩极其不易。这是省委、省政府和市委正确领导的结果，是市人大依法监督、市政协民主监督、社会各界大力支持的结果，是全市人民团结奋斗、顽强拼搏的结果。在此，我代表市人民政府，向全市广大工人、农民、知识分子和各级干部，向驻汴人民解放军指战员、武警官兵、公安民警，向关心和支持政府工作的各民主党派、工商联、各人民团体和社会各界人士，表示崇高的敬意和衷心的感谢！

在肯定成绩的同时，我们也清醒地看到，我市经济社会发展和政府工作中还存在一些不容忽视的问题。主要是：经济回升的基础还不牢固，财政收支压力增大，就业形势依然严峻；经济总量仍然偏小，自主创新能力不强，结构调整任重道远；农业基础仍较薄弱，保持粮食稳定增产、农民持续增收难度增加；一些涉及群众切身利益的问题还没有解决好，保障和改善民生的机制尚不健全，保障水平还不高；安全生产、重大疫情防控、维护社会稳定等任务还相当艰巨；干部作风有待进一步转变，政府效率、执行能力亟待提高。对此，我们一定高度重视，采取有效措施，认真加以解决。

二、2010年政府工作的总体要求和奋斗目标

今年是实施“十一五”规划的最后一年，也是应对国际金融危机的关键之年，做好今年的政府工作，对奋力实现开封经济社会发展全面跨越，具有十分重要的意义。总体上看，今年的发展形势要明显好于去年，世界经济出现企稳回升的积极迹象；我国经济整体向好态势不断巩固，国家继续实施积极的财政政策和适度宽松的货币政策，在保持经济平稳较快发展的同时，加快经济结构调整，促进经济发展方式转变，鼓励自主创新，扩大民生投入，这些都为我们加快发展提供了有利条件。特别是省委、省政府对开封发展寄予厚望，强力推进郑汴新区建设，打造经济社会发展核心增长极，为我们加快发展、跨越式发展指明了方向。当前，宏观经济形势仍然比较复杂，我市经济持续增长的内生动力不足，扩大需求存在较大制约，经济结构性矛盾还很突出，总量不大、层次不高、实力不强、后劲不足等问题并存。面对复杂多变的经济形势，我们既要坚定必胜信心，又要增强忧患意识，进一步振奋精神，奋起争先，牢牢把握重要战略机遇期，努力在新的起点上把改革、发展、稳定各项工作做得更好。

今年政府工作的总体要求是：全面贯彻党的十七大和十七届三中、四中全会精神，以邓小平理论和“三个代表”重要思想为指导，深入贯彻落实科学发展观，坚持重在持续、重在提升、重在统筹、重在为民，坚定不移地实施工业强市、开放引进、旅游带动、科教兴汴、城市化和可持续发展战略，着力推进经济结构调整，转变经济发展方式；着力推进工业化、城镇化、农业现代化，实现“三化”协调发展；着力深化改革和扩大开放，增强经济发展的动力和活力；着力保障和改善民生，保持社会和谐稳定，加快实现老工业基地向新型工业化城市跨越、传统农业向现代农业跨越、文化旅游大市向文化旅游强市跨越，全面开创经济社会跨越式发展新局面。

综合考虑各种因素，今年经济社会发展主要预期目标为：生产总值增长12%以上；地方财政一般预算收入增长12%以上；全社会固定资产投资增长28%；社会消费品零售总额增长17%；外贸出口增长11%；实际利用外资增长18%；居民消费价格涨幅3%；城镇居民人均可支配收入实际增长7%；农民人均纯收入实际增长6%；人口自然增长率控制在6.5‰以内；城镇新增就业6万人，城镇登记失业率控制在4.5%以内；单位生产总值能耗降低4.76%；化学需氧量、二氧化硫排放量分别下降3.4%和1.1%；发展方式转变和经济结构调整取得重大进展。

三、奋力实现经济社会发展全面跨越

完成今年各项目标任务，必须在巩固经济回升态势的基础上，针对我市经济社会发展中的薄弱环节，创新思路，创新举措，创新工作，努力促进经济社会科学发展、和谐发展、跨越式发展。

（一）着力实施“八大工程”和“八大行动计划”，推进经济社会发展全面跨越。“八大工程”即产业集聚区建设工程、转型升级“3135”工程、现代城镇体系建设工程、自主创新体系建设工程、十五项民生工程、开封新区建设工程、新农村建设“百村示范”工程、文化改革发展试验区建设工程；“八大行动计划”即重点改革行动计划、企业服务行动计划、国企改革攻坚行动计划、就业促进行动计划、对外开放行动计划、粮食稳产保收行动计划、人力资源素质提升行动计划、安全发展和平安建设行动计划。“八大工程”和“八大行动计划”是今年工作的重中之重，是完成全年目标任务的主要载体，必须精心谋划，务求实效。突出抓好项目建设。把项目建设当作促进经济社会发展全面跨越的重要抓手，以大项目带动大投资，以大投资引领大发展。强力实施转型升级“3135”工程，实施300个重大建设项目，带动项目总投资1000亿元以上，当年完成投资额突破300亿元，全年实现全社会固定资产投资突破500亿元。千方百计扩大投资。结合“十二五”规划编制，在工业、农业、能源、生态建设、产业集聚区、现代服务业、民生工程等方面，高起点谋划一批重大项目，为经济发展备足后劲。积极争取国家、省在资金、项目、政策方面的支持，确保投资持续增长。进一步改善投资环境，拓宽民间投资渠道，吸引社会资本进入教育、卫生、文化和基础设施等领域，努力形成多元化、社会化的投资格局。加大金融支持力度。进一步优化金融生态环境，加强与各金融机构的沟通，积极组织银企对接，促进银企合作，确保年内新增贷款80亿元以上。着力加快金融业发展，创造条件，吸引股份制银行在开封设立分支机构；积极发展村镇合作银行，破解农村金融市场发展相对滞后的局面；充分发挥中小企业投资担保机构的作用，增强担保贷款能力。积极推进企业上市，全力支持德意环保、新大新、平原非标等企业上市融资。

（二）着力加快工业经济发展，为跨越式发展提供战略支撑。坚持把推进新型工业化作为跨越式发展的根本，着力壮大企业群体、优化工业结构、增强工业实力，提升工业经济发展质量和水平。继续搞好企业服务。实施“企业服务行动计划”，建立健全政策导向、要素保障、环境优化、融资服务、困难企业帮扶等十项长效机制，推动企业服务常态化。继续抓好重点服务企业并实行动态管理，把服务企业与行风政风评议结合起来，切实提高服务效果。大力推进工业结构调整。围绕国家和省十大产业调整振兴规划，高起点、高标准制定我市优势行业和新兴产业发展规划。积极推进汽车及零部件、装备制造、精细化工等产业集聚，带动全市产业布局优化、结构升级。积极培育新兴产业，鼓励我市企业与国内外大型企业开展战略合作，通过招商引资、升级改造、政策扶持，催生和壮大新材料、光电、光伏、生物医药等一批面向未来的战略支撑产业。狠抓重大工业项目建设。全力推进河南煤化集团聚甲醛、浙江东磁集团单晶硅、晋开集团百万吨总氨、奇瑞汽车及零部件、河南煤化重装集团及空分基地、中平能化光气产业园等重大工业项目的实施。着力培育重点企业。深入实施“重点企业培育工程”，落实领导分包重点企业制度，加大重点企业扶持力度，及时协调解决重点企业发展中的困难和问题，帮助企业做强做大，促进企业转型升级，争取有2－3家企业主营业务收入超过20亿元。抓好工业经济运行调度。加强运行监测和要素供需协调。密切关注国家宏观调控政策和市场变化，及时研究实施针对性政策措施。加强煤电油气运供需调度，确保正常生产生活需要。

（三）着力巩固“三农”发展基础，推进社会主义新农村建设。坚持把解决好“三农”问题作为全部工作的重中之重，巩固提高粮食综合生产能力，大力发展现代农业，进一步夯实“三农”发展基础。巩固提高粮食综合生产能力。实施“粮食稳产保收行动计划”，强力推进粮食核心区建设，认真抓好优质粮产业工程、沃土工程、测土配方施肥等重大技术项目，力争全年粮食总产51亿斤。强化抗灾应急能力建设，建立市、县（区）引黄灌溉水费保障机制。按期完成赵口、柳园口、三义寨三个大型灌区续建配套与节水改造工程和运粮河治理工程年度建设任务，对铁底河、黄汴河等10条骨干河渠进行清淤疏浚。大力发展现代农业。积极推进农业结构调整，继续抓好优质粮食、棉花、花生、瓜菜4个百万亩优质农产品产业带建设。全面实施现代畜牧产业发展规划，加快推动优质畜产品生产加工基地建设和规模养殖场标准化改造升级。继续强化重大动（植）物疫病防控和农畜产品质量安全监管。积极培育农业产业化龙头企业，大力发展农民专业合作组织，创建30个市级农民专业合作社示范社，扶持15个农机专业合作社，不断提高农业组织化程度。发展壮大县域经济。立足县域资源优势和产业基础，加大招商引资力度，引进一批有利于优化经济结构、增强发展后劲的重大项目，培育一批具有较强带动作用的龙头企业，发展壮大县域支柱产业，提升县域产业发展水平。全面落实支持县域经济发展的各项政策，突出发展民营经济，大力发展农村二、三产业，促进县域经济晋位升级。积极促进农民增收。继续落实好对农民的各项补贴政策，较大幅度提高农资综合直补、良种补贴和农机具购置补贴标准。认真落实小麦、稻谷等主要粮食品种最低收购价政策。积极推进扶贫开发，完成55个扶贫开发整村推进村、4.9万贫困人口脱贫任务。继续实施“阳光工程”、扶贫培训“雨露计划”和“农村劳动力技能培训计划”，加大农村劳动力技能培训力度，全年转移农村劳动力87.5万人，其中新增转移就业农村劳动力6万人。扎实推进新农村建设。实施社会主义新农村建设“百村示范”工程，确定100个新农村示范村，对20个示范中心村、80个示范村给予补助和奖励。增加并整合支农资金，加强农村基础设施建设与监管，修建县乡公路200公里、通村公路100公里，解决20万农村居民安全饮水问题，新增户用沼气1.5万户，改建农村卫生厕所1.1万座。继续实施“万村千乡市场工程”，再建设400个农村连锁超市，覆盖全市80%以上行政村。通过示范带动，分类指导，强力推进，努力开创社会主义新农村建设新局面。

（四）着力发展文化旅游产业，推进文化旅游大市向文化旅游强市跨越。实施“文化改革发展试验区建设工程”，充分挖掘开封文化内涵，发挥优势，培育精品，主打宋文化品牌，做大做强文化旅游产业。加快整合文化旅游资源。紧紧抓住我市被确定为省文化产业示范园区和省文化改革发展试验区的重大机遇，进一步理顺体制，创新机制，争创国家级文化产业示范园区。以市文化旅游投资集团有限公司为平台，整合文化旅游资源，拓宽投融资渠道，通过集团化运作，形成以产业集团为骨干、各类文化旅游企业竞相发展的文化旅游产业集群，提高文化旅游产业整体竞争力。强化项目谋划和建设。继续深入实施宋都古城风貌保护与重现工程，认真抓好水系工程、城墙保护与综合整治、繁塔·禹王台景区、大宋文化博物馆·开封城摞城新郑门遗址等重大项目建设；着力谋划和包装一批重大文化旅游项目，启动延庆观景区开发、刘家胡同古街区、大河动漫城、中原明珠广播电视塔文化广电基地等项目建设，推动文化旅游产业快速发展。精心打造文化旅游品牌。继续抓好大型水上实景演出《大宋·东京梦华》等著名文化品牌建设，不断扩大龙亭湖风景区等旅游品牌影响力；认真办好第十届中国菊花展览会暨中国开封第28届菊花花会、2010年中国开封清明文化节、第二届中国木版年画艺术节等一系列文化节会活动，努力形成一批特色鲜明的宋文化品牌。加强文化旅游推介。培育新型文化旅游业态和产品，采取多种形式，加大文化旅游推介力度，大力开拓文化旅游市场，提升开封文化旅游知名度和影响力。

（五）着力抓好产业集聚区建设，加快推进城镇化进程。深入实施“产业集聚区建设工程”和“现代城镇体系建设工程”，按照“企业集中布局，产业集群发展，资源集约利用，功能集合构建”的总体要求，加快产业集聚区发展，努力实现产城互动、融合发展。加快规划编制与实施。尽快编制完成各产业集聚区控制性详规和修建性详规。实施产业集聚区投资倍增计划，加快道路、供电、供气、污水处理、标准化厂房等基础设施建设，力争完成投资240亿元。加快公共基础设施和服务设施建设，引导学校、医院、服务网点等配套服务机构向产业集聚区聚集，提高产业集聚区承载能力。创新管理体制和运营机制。理顺开封新区和各产业集聚区管理体制，建立健全产业集聚区发展考核体系。探索建立基础设施建设、土地保障、投融资、自主创新、高效管理和社会化服务等有利于促进产业集聚区发展的机制，出台电价、财税、土地、环保、资金等支持产业集聚区建设的政策，促进产业集聚区协调快速发展。健全完善产业集聚区投融资平台，力争每个投融资平台资产规模超过1亿元，全年融资不低于1.2亿元。加快县城建设步伐。以产业集聚区建设为载体，着力加快县城建设。按照高起点规划、高标准建设、高水平管理的要求，深化和完善县城规划，提升县城规划水平，严格规划管理；加大县城开发建设力度，进一步拉大城市框架，扩大县城规模；加强县城管理，提高县城精细化管理水平，着力提升县城品位。同时，积极抓好小城镇建设，开展中心镇建设试点工作，每县至少抓好1个中心镇；突出抓好朱仙镇开发建设，重现历史文化名镇风采。

（六）着力加快开封新区建设，打造经济社会发展核心增长极。开封新区承载着开封经济社会发展全面跨越的使命和希望，是开封经济社会发展的强力引擎。要从一切有利于新区发展的角度出发，举全市之力支持开封新区建设，着力打造全市经济社会发展核心增长极。进一步完善规划。制订和完善运粮河组团城市设计方案，尽快编制运粮河组团修建性详规，争取下半年启动运粮河组团基础设施建设。尽快编制铁路以南工业区建设规划，启动铁路以南排水管网和道路建设，为项目入驻创造条件。全面加快基础设施建设。加快新区道路、管网、通讯及生活服务设施建设，使新区基本具备生产生活条件，提高新区的吸引力和承载力。加快新区一期、二期道路建设；投入30亿元，实施新区三期、四期和基础设施BT项目建设；开工建设东京桥、金耀桥、晋安桥、郑开桥和众意湖景观桥。加快郑汴物流通道开封段、黄河大堤道路加固、连霍高速开封段拓宽、郑民高速开封段、郑州至开封城际铁路建设。迅速掀起项目建设高潮。将2010年确定为开封新区项目建设年，加快推进奇瑞汽车制造基地、河南煤化重装集团、大河动漫城等重大项目建设。加快职业教育园区和黑岗口调蓄水库建设；启动香港爪哇集团大型综合购物广场建设。积极申请建立国家级空分特色产业基地，争创国家级经济技术开发区。强力推进迁村并居。加快闫砦、毛砦，野厂、瞿家砦迁村并居工程建设步伐；启动横堤铺、王府寨、斗门迁村并居工程。

（七）着力深化改革开放，为跨越式发展增添动力和活力。实施“重点改革行动计划”、“国企改革攻坚行动计划”，着力构建充满活力、富有效率、有利于科学发展的体制机制。深化行政管理体制改革。积极推进政府机构改革，确保一季度完成市县政府机构改革。继续压缩和规范行政审批事项，强力推行涉企收费“一费制”和项目代理制，提高办事效率和服务水平，进一步优化经济发展环境。规范、完善财政转移支付制度，健全县级基本财力保障机制，推进基本公共服务均等化。深化事业单位改革。积极推进事业单位分类改革和事业单位岗位管理制度改革。深化教育、卫生系统事业单位人事制度改革，全面推行全员聘用和绩效工资制度。继续深化交通运输体制改革，做好二级公路收费站收费人员安置工作。加快文化旅游体制改革。全面完成经营性文化事业单位转企改制，启动市杂技团、豫剧院转企改制。深化公益性文化事业单位内部三项制度改革，加快推进经营性报纸、期刊、出版单位转企改制，全面展开电台电视台制播分离改革。深化旅游景区管理体制改革，市文化旅游投资集团有限公司开展实质性运营。进一步深化企业改革。加快推进中州机械、羊洋集团、亚太啤酒公司、博达集团等企业产权制度改革和破产重组；做好开化集团、联合收割机厂、柴油机厂等企业破产改制的收尾工作；加强对已改制企业的履约监管，巩固企业改革成果。同时，加快推动收入分配、资源价格、环保收费等领域改革。着力提高开放型经济水平。实施“对外开放行动计划”，积极开展“千名人员专业大招商活动”；推进“双百工程”，推动2009年签约的100个亿元以上重大招商引资项目落地，争取新签约亿元项目100个以上。承办好河南省第五届豫商大会。以承接产业转移、加强与央企合作、搞好技术人才引进为重点，坚持以企业和县区为主、民间和商会为主、专题对口活动为主，切实提高招商引资活动的针对性。进一步优化产品结构，推进外贸出口稳定增长。支持具备条件的企业“走出去”，大力发展对外劳务合作；鼓励企业到境外承包工程，带动成套设备、技术、服务和国际劳务输出。

（八）着力推进发展方式转变，增强可持续发展能力。把转变发展方式摆在更加突出的位置，不断强化自主创新能力，务求在一些重要领域和关键环节实现突破。切实提升自主创新能力。全面落实自主创新体系建设规划，实施“自主创新体系建设工程”，积极培育具有自主创新能力的高新技术企业，提升企业自主创新能力和高新技术产业规模。做好茂盛机械有限公司“国家粮食加工装备工程技术研究中心”组建工作，争取再申报1家国家级工程技术研究中心。积极拓展国际科技合作领域，抓好国家超级小麦育种、国际农业高新技术产业园等基地建设。深化与中国农业大学、河南大学等院所的合作，启动中国农业大学开封实验站建设。创新政府资金支持机制，重点用于科研项目成果转化应用和高层次人才引进培养。推动节约集约发展。完善节能减排体制机制，进一步提高资源利用效率，积极发展循环经济和低碳经济。坚持最严格的耕地保护制度和最严格的节约用地制度，大力推进土地综合整治，积极挖掘用地潜力，严禁违法用地和闲置用地，控制粗放用地。强化节能降耗，突出抓好工业、交通、建筑等关键领域节能工作，加快淘汰行业落后产能。严把新建项目准入关，严格“两高一资”项目审查，对能耗指标达不到国内同行业先进水平的新建和改扩建项目不予批准建设。加快节水工程建设，推进节水技术改造。开展自备井限采封存集中整治行动，依法加强对我市地下水资源的管理，合理开发、利用和保护水资源。大力开展节能减排全民行动，在全市形成节能减排浓厚氛围。强化环境保护和生态建设。继续加强对惠济河等重点流域，化工、化肥、制药、制革等重点行业和柳池、黑池等饮用水源保护区环境综合整治；建成全市环境自动监控系统；加快垃圾处理场和污水处理厂管网配套建设，开工建设污水处理厂中水回用和污泥处置工程。开展重点排污企业强制性清洁生产审核，按照清洁生产的要求，加强对建设项目的监督管理。大力发展节能环保产业。加强农村污染治理。继续推进林业生态市建设，完成造林面积10.3万亩。

（九）着力实施民生工程，促进经济社会协调发展。坚持把民生工作摆在更加突出的位置，统筹安排好各项民生工程，继续办好15件为民实事，让改革发展成果惠及全市人民。继续做好就业和再就业工作。实施“就业促进行动计划”，确保全市全年城镇新增就业人员6万人，失业人员再就业2.5万人，其中帮助就业困难人员就业0.9万人，动态消除“零就业家庭”。不断提高社会保障水平。继续提高企业退休人员养老金标准，幅度不低于全市平均养老金水平的10%；搞好通许县新型农村社会养老保险试点工作；进一步完善城乡低保制度，适当提高城乡低保标准；深入推进新农合制度，参合率达到95%，参合标准提高到每人每年120元。大力实施保障性安居工程。新开工建设经济适用住房30万平方米、竣工30万平方米，为城市低收入住房困难家庭提供3000套以上经济适用住房；开工建设廉租住房3500套；加大廉租住房租赁补贴发放力度，年底前完成保障户数1.1万户；加快农村危房改造。优先发展教育事业。实施“人力资源素质提升行动计划”，努力把人口压力转化为人力资源优势。推进义务教育均衡发展，继续提高义务教育经费保障水平，对全市农村中小学生和城市低保家庭学生免费提供教科书，为农村家庭经济困难寄宿生提供生活费补助；在新区新建3所小学，启动开封高中西校区建设，缓解新区学生入学难问题；更加关注农村留守儿童教育问题，着力解决进城务工人员随迁子女入学和城市大班额问题；扎实推进中小学校舍安全工程，全市义务教育阶段学校达标率再提高25个百分点。理顺职业教育管理体制，扎实推进职业教育攻坚计划，继续为中等职业学校学生发放国家助学金，免除中等职业学校家庭经济困难和涉农专业学生学杂费；加快开封大学新校区和省示范性高职院校建设，全力推进开封文化艺术职业学院建设。建成市委党校综合报告厅。大力发展卫生事业。进一步加强医疗卫生服务体系建设，加大基层医疗卫生人才培养力度，加快医疗卫生资源整合步伐；启动新区中心医院建设，迁建市精神病医院，建成市中医院病房楼并投入使用；加强农村合作医疗资金监管，着力解决群众看病难、看病贵问题；继续搞好甲型H1N1流感等重大传染病防控工作。积极做好人口和计划生育工作。继续实施计划生育家庭特别扶助制度和农村部分计划生育家庭奖励扶助制度；全面启动优生促进工程，提高出生人口素质；开展“关爱女孩”行动，综合治理出生人口性别比偏高问题。加快城市基础设施建设。完成龙亭湖周边环境综合整治、黄河大街北延、五福路改造、西环路北延、东环路北延、劳动路北延、东京大道东延和东区排水沉陷点改造工程建设，开工建设复兴大道。加快老城区改造步伐，全面实施老城区1号、2号地块改造与开发，积极推进“城中村”和城市棚户区改造。升级改造夜市和农贸市场，擦亮城市窗口。进一步加强社区建设。改（扩）建社区办公场所33个、社区卫生服务中心5个；继续提高社区工作人员待遇，整合完善社区服务功能，提高社区服务水平。努力保持社会大局稳定。实施“安全发展和平安建设行动计划”，深入开展重点领域隐患治理，建立安全生产投入保障机制，严格市场准入和退出机制，遏制重特大事故发生。整顿食品药品市场，严厉打击非法生产经营，确保食品药品安全。深入推进社会矛盾化解、社会管理创新和公正廉洁执法建设，坚决纠正损害群众利益的突出问题，妥善处理土地征用、拆迁安置、企业改制、环境污染、劳资纠纷、涉法涉诉等方面的矛盾和问题；积极预防和妥善处置群体性事件和突发公共事件；加强和改进信访工作；注重解决好社会治安问题较多的城区和乱点问题，健全社会治安防控体系，依法防范和严厉打击各类违法犯罪活动，确保社会大局和谐稳定。加强精神文明和民主法制建设。深入推进社会主义核心价值体系建设，大力弘扬以爱国主义为核心的民族精神和以改革创新为核心的时代精神。积极开展文明单位结对帮扶和城乡共建精神文明活动，深化文化科技卫生“三下乡”和“四进社区”活动，推动群众性精神文明创建活动蓬勃开展。加强精神文明教育，提高市民文明意识和交通安全意识。继续开展全民健身活动，办好2010年郑开国际马拉松赛，积极迎战十一届省运会。加强基层民主建设，进一步完善村民自治。加强法律援助，努力做到应援尽援。同时，继续加强国防教育和国防后备力量建设，支持驻汴解放军和武警部队建设；巩固发展军政军民团结，再创全国双拥模范城。支持工会、共青团、妇联等人民团体发挥桥梁纽带作用，积极参与社会管理和公共服务。认真做好第六次人口普查工作。继续做好外事、侨务、对台、民族、宗教、统计、史志、人防、气象、地震、社科研究等工作。全面完成江油援建任务。认真编制好“十二五”规划。

（十）着力提高经济社会管理水平，建设人民满意的政府。以建设城市数字化管理中心为契机，切实提高政府管理经济社会的能力和水平，努力建设为民、责任、务实、高效、廉洁政府。建设城市数字化管理中心。用三年时间、分三期工程，建设覆盖全市建成区，涉及道路交通、公用设施、市容环境、园林绿化、建（构）筑物、突发性公共事件、社会服务、企业服务等管理领域，涉及城管、工商、公安、旅游、国土、建设、交通、环保、人防、安全生产、市场发展等管理对象的高标准、高质量、高效率数字化城市管理信息系统，建立精确、敏捷、高效、全时段、全方位覆盖的城市管理新模式，为人民群众提供更加优质、便捷、高效的服务。今年启动一期工程建设，力争9月底前城市数字化管理中心建成投用，形成“一级监督、两级指挥、三级管理、四级网络”的城市管理新机制。切实加强政府自身建设。深入开展学习实践科学发展观活动，扎实推进“两转两提”，进一步转变政府职能，转变工作作风，着力提高政府的公信力和执行力。坚持以人为本，执政为民，真正做到权为民所用、情为民所系、利为民所谋，推进服务型政府建设。坚持科学决策，民主决策，严格执行人大及其常委会的决议、决定，自觉接受人大法律监督和政协民主监督，充分听取各民主党派、工商联、无党派人士及各人民团体的意见，广泛接受社会公众和新闻舆论监督，推进责任政府建设。坚持求真务实，真抓实干，少提口号、多办实事，坚决克服形式主义、官僚主义，推进务实政府建设。坚持依法行政，从严治政，着力提高工作效率，做到政令畅通、令行禁止，推进效能政府建设。坚持标本兼治、综合治理、惩防并举、注重预防的方针，严格落实党风廉政建设责任制，完善惩治和预防腐败体系，强化对行政权力运行的监督制约，推进廉洁政府建设。

各位代表！

2010年是开封经济社会发展全面跨越的关键之年，也是全面完成“十一五”规划、奠定“十二五”发展基础的重要一年。目前，我市经济发展处于上升期，产业结构处于调整期，各项改革处于攻坚期，我们面临的任务艰巨而繁重，我们肩负的使命重大而光荣。我们必须进一步增强忧患意识、机遇意识和竞争意识，以跨越发展的虎虎生气，带领全市人民，紧密团结在以胡锦涛同志为总书记的党中央周围，高举中国特色社会主义伟大旗帜，坚持以邓小平理论和“三个代表”重要思想为指导，深入贯彻落实科学发展观，在省委、省政府和市委的坚强领导下，开拓创新，顽强拼搏，为实现开封经济社会发展全面跨越而努力奋斗！
